# Inflammatory Bowel Disease and Stroke: Exploring Hidden Vascular Risks

**DOI:** 10.7759/cureus.79304

**Published:** 2025-02-19

**Authors:** Abdallah Khan, Maysoon A Azzam

**Affiliations:** 1 Internal Medicine, RAK Medical and Health Sciences University, Ras Al Khaimah, ARE

**Keywords:** crohn's disease, inflammation, inflammatory bowel diseases, stroke, ulcerative colitis

## Abstract

Inflammatory bowel disease (IBD), encompassing Crohn’s disease and ulcerative colitis, is primarily known for its gastrointestinal manifestations. However, emerging evidence suggests a potential link between IBD and an increased risk of stroke, likely mediated by chronic systemic inflammation, endothelial dysfunction, and a prothrombotic state. Despite this growing recognition, the exact mechanisms and extent of this association remain unclear, highlighting a critical knowledge gap. This review aims to systematically analyze the association between IBD and stroke, exploring the underlying vascular mechanisms and identifying potential risk factors contributing to cerebrovascular events in IBD patients. A comprehensive literature search was conducted following the Preferred Reporting Items for Systematic Reviews and Meta-Analyses (PRISMA) guidelines across PubMed, Scopus, and Google Scholar using keywords such as “IBD,” “Stroke,” “Chronic inflammation,” “Cerebrovascular risk,” and “Gut-brain axis.” After screening 150 studies and applying inclusion and exclusion criteria, six studies were included in the final synthesis. The findings suggest that chronic inflammation in IBD plays a key role in increasing stroke risk through endothelial dysfunction and a heightened prothrombotic state, with additional risk factors such as atrial fibrillation during active IBD flares further contributing to cerebrovascular events. While biologic therapies, including tumor necrosis factor (TNF)-alpha inhibitors, are effective in reducing systemic inflammation, their impact on mitigating stroke risk remains inconclusive. Given the potential role of IBD as an independent risk factor for stroke, a multidisciplinary approach to management is crucial. Addressing modifiable risk factors through pharmacologic interventions such as biologics, statins, and antiplatelet agents, alongside lifestyle modifications, could help reduce cerebrovascular complications in IBD patients. Further research is needed to explore personalized therapeutic strategies and establish clearer preventive guidelines for this at-risk population.

## Introduction and background

An increasingly common chronic inflammatory illness of the gastrointestinal (GI) tract worldwide is inflammatory bowel disease (IBD); it includes ulcerative colitis (UC) and Crohn's disease (CD) [1]. Over the past several decades, IBD incidence and prevalence have gradually increased worldwide, especially in recently industrialized nations [1]. For example, it is estimated that about 400 per 100,000 people in North America and Europe have IBD [1]. Owing to its chronic nature and increasing occurrence, IBD's extraintestinal symptoms, such as a higher risk of cardiovascular events like stroke, have received more attention [1].

Stroke is one of the leading causes of death and disability worldwide [2], with over 12 million new strokes occurring each year and a global mortality rate exceeding 6.5 million [3]. About 87% of all stroke cases are ischemic strokes, which are brought on by arterial obstruction, while the remaining stroke cases are hemorrhagic strokes, which are typically caused by ruptures in the vessels [4]. Stroke has a high death rate, but it also causes long-term impairment that has a high social and economic cost [4]. The increasing prevalence of stroke worldwide emphasizes how crucial it is to discover new risk factors and comprehend the underlying pathophysiological processes [4].

Although IBD primarily affects the GI system, an increasing amount of evidence suggests that systemic inflammation characteristic of IBD might play a critical role in promoting cerebrovascular diseases, including stroke [5]. The relationship between IBD and stroke risk has been the subject of multiple studies, although the findings are still inconclusive [6]. However, according to research, individuals with IBD have a noticeably higher risk of stroke [7]. Additionally, the presence of comorbid conditions in individuals with IBD further elevates the risk [8]. Systemic inflammation, oxidative stress, and a prothrombotic state are standard features of IBD that may exacerbate endothelial dysfunction and raise the possibility of cerebrovascular events [9]. Moreover, the association is further complicated by medications like corticosteroids used to treat IBD, as they can significantly increase conventional stroke risk factors such as dyslipidemia and hypertension [10].

This article explores the association between IBD and the elevated risk of stroke, focusing on the underlying mechanisms and factors that link these two conditions. By examining how IBD contributes to increasing the risk of stroke, this analysis offers valuable insights into the management of patients with both diseases. Understanding this relationship will aid in improving stroke preventive strategies and enhancing patient outcomes for IBD patients.

## Review

Methods

Search Strategy

**Figure 1:**
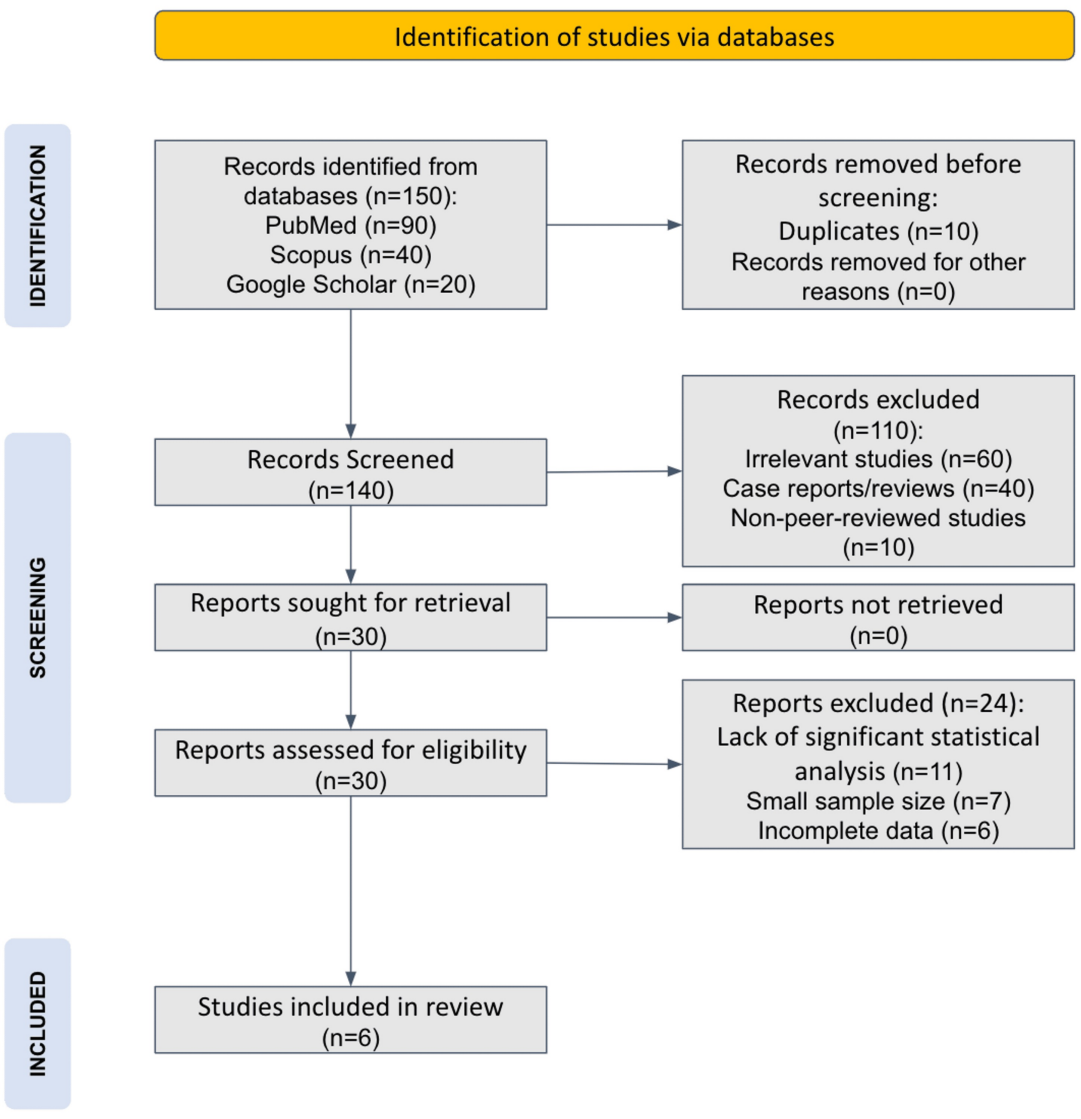
PRISMA Flowchart of Selected Articles PRISMA: Preferred Reporting Items for Systematic Reviews and Meta-Analyses

The authors (AK and MA) conducted a comprehensive review following the Preferred Reporting Items for Systematic Reviews and Meta-Analyses (PRISMA) guidelines. A comprehensive search was performed in databases, including PubMed, Scopus, and Google Scholar, using keywords such as “IBD,” “Stroke,” “Chronic inflammation,” “cerebrovascular risk,” and “gut-brain axis.” Boolean operators (AND, OR) were used to refine the search results. The initial search identified 150 articles, of which 140 remained after duplicates were removed. Titles and abstracts were screened for relevance, and 30 full-text articles were further assessed for eligibility. After applying the inclusion and exclusion criteria, six studies were included in the final synthesis. The study selection process is detailed in the PRISMA flowchart (Figure 1).

Inclusion and Exclusion Criteria

Inclusion criteria targeted observational studies (cross-sectional, cohort, and meta-analyses) published in English between January 2008 and December 2024 that investigated the association between IBD and stroke. Studies focusing on vascular implications, chronic inflammation, and the gut-brain axis were included by the authors (AK and MA).

Exclusion criteria followed PRISMA recommendations, excluding non-English studies, case reports, editorials, and reviews without primary data (n=40). Full-text articles were excluded if they were statistically insignificant (p>0.05) (n=11), had small sample sizes (less than 500 patients) (n=7), or presented incomplete or unclear data (n=6). The selection process, including screening and final study inclusion, was independently conducted by the authors (AK and MA).

Results

The search yielded 150 articles. Following PRISMA-guided screening, 110 were excluded due to irrelevance (n=60), being case reports or reviews without primary data (n=40), or being non-peer-reviewed or qualitative studies (n=10). Of the 30 full-text articles assessed, 24 were excluded for lack of statistical analysis (n=11), small sample size (n=7), or incomplete data (n=6). Finally, six studies were included in the review. The detailed process is summarized in the PRISMA flowchart (Figure 1).

Navigating the pathophysiological overlap between IBD and stroke

Chronic Intestinal Inflammation

Dysregulated immune responses contribute to the development of IBD, which is characterized by chronic inflammation of the GI tract and includes CD and UC [11]. Environmental triggers such as diet, infections, and gut microbiota dysbiosis in genetically predisposed individuals initiate and perpetuate this aberrant immune response [12]. The immune system in IBD patients exhibits a hyperactive response to luminal antigens, resulting in the overproduction of cytokines that promote inflammation, such as tumor necrosis factor-alpha (TNF-alpha), interleukin-1 (IL-1), IL-6, and IL-12 [13].

Disruption of Epithelial Barrier Function

A key feature of IBD is the compromised integrity of the intestinal epithelial barrier, which generally limits the translocation of luminal antigens and pathogens [14]. Due to the lack of tight junction proteins and increased intestinal permeability, microbial products can pass through the lamina propria, further triggering immune activation [15]. This barrier dysfunction is more pronounced in CD, where deeper, transmural inflammation occurs, compared to superficial inflammation in UC [16].

Activation of Innate and Adaptive Immunity

The innate immune system, involving neutrophils, macrophages, and dendritic cells, becomes hyperactivated in IBD [17]. These cells and cytokines produce reactive oxygen species (ROS) damaging tissues [17]. Additionally, the adaptive immune response drives chronic inflammation through sustained cytokine release, particularly T-helper (Th1, Th17) cells in CD and T-helper 2 cells in UC [18]. IBD frequently results in malfunctioning regulatory T cells (Tregs), which regulate immunological responses and contribute to uncontrolled inflammation [19].

Role of Microbiota

Gut dysbiosis, or the imbalance of the intestinal microbiome, significantly influences the pathophysiology of IBD [20]. In IBD patients, there is a reduction in protective bacterial species such as *Faecalibacterium prausnitzii* and a surge in pathogenic bacteria like *Escherichia coli* [20]. This dysbiosis contributes to a pro-inflammatory state by promoting immune system activation and a breakdown in mucosal tolerance [21].

Genetic Predisposition

Several genetic mutations have been linked to IBD, including mutations in the nucleotide-binding oligomerization domain-containing protein 2 (NOD2) gene, which plays a role in microbial recognition and immune response [22]. Mutations in genes regulating autophagy, such as autophagy-related 16 like 1 (ATG16L1), and those involved in maintaining the epithelial barrier, like the mucin 2, oligomeric mucus/gel-forming (MUC2), also predispose individuals to IBD [23]. The abnormal immune responses observed in IBD result from these hereditary variables [20].

Systemic Immune Activation

While inflammation is primarily localized to the GI tract, IBD is associated with systemic immune activation, which can affect other organs, including the cardiovascular system [24]. This systemic inflammation is marked by elevated levels of circulating cytokines and acute-phase proteins such as C-reactive protein (CRP), which are implicated in vascular complications [25]. The chronic inflammatory state also promotes endothelial dysfunction and oxidative stress, linking IBD to increased vascular risks [26].

**Figure 2:**
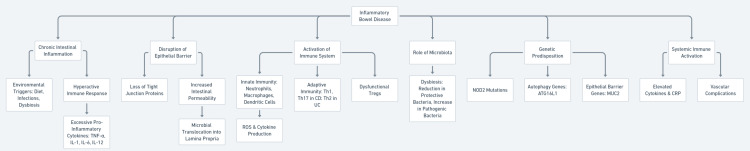
Pathophysiological Mechanisms Underlying Inflammatory Bowel Disease TNF-α: tumor necrosis factor-alpha; IL-1: interleukin-1; IL-6: interleukin-6; IL-12: interleukin-12; CD: Crohn’s disease; UC: ulcerative colitis; Th1: T-helper 1 cells; Th17: T-helper 17 cells; Th2: T-helper 2 cells; ROS: reactive oxygen species; CRP: C-reactive protein; NOD2: nucleotide-binding oligomerization domain-containing protein 2; ATG16L1: autophagy-related gene 16-like 1; MUC2: mucin 2 Image credits: The image has been created by the author Maysoon A. Azzam.

Patients with IBD, including CD and UC, are at an increased risk of both arterial and venous thromboembolic events, including stroke [26]. The pathophysiology linking IBD to stroke involves chronic systemic inflammation, endothelial dysfunction, and a hypercoagulable state [27]. These are summarised in Figure 2.

Chronic Systemic Inflammation

A chronic systemic inflammatory state is characteristic of IBD [1]. Cytokines like TNF-alpha, IL-6, and CRP are chronically elevated, contributing to endothelial dysfunction and promoting atherosclerosis, a primary driver of ischemic stroke [27]. The continuous activation of the immune system in IBD leads to a heightened inflammatory response, which affects the gut and results in systemic inflammation [28]. This systemic inflammation extends beyond the GI tract and is implicated in the development of cardiovascular diseases, including stroke [28]. Elevated cytokines, such as IL-1β, IL-8, and TNF-alpha, are essential contributors to endothelial damage and the upregulation of pro-atherogenic factors like vascular cell adhesion molecule 1 (VCAM-1), which promotes leukocyte recruitment to endothelial surfaces, triggering vascular injury and atherosclerosis [29].

Endothelial Dysfunction and Atherosclerosis

IBD leads to chronic endothelial dysfunction, a critical factor in the development of atherosclerosis and, consequently, ischemic stroke [30]. By controlling mechanisms, including coagulation, vasodilation, and immunological responses, endothelial cells contribute significantly to vascular homeostasis [30]. Endothelial dysfunction in individuals with IBD is caused by oxidative stress and a reduction in the bioavailability of nitric oxide (NO), a vital vasodilator [30]. Reduced antioxidant defenses and elevated ROS aggravate oxidative stress in IBD patients, resulting in endothelial dysfunction and damage [31]. Over time, this dysfunction promotes the development of atherosclerotic plaques [32]. Plaque rupture or erosion can result in arterial thrombosis, causing an ischemic stroke [32]. Furthermore, during periods of active IBD, inflammatory markers are elevated, which can destabilize atherosclerotic plaques and increase the risk of thromboembolic events [33].

Hypercoagulability and Thrombosis

IBD patients are in a hypercoagulable state, increasing their susceptibility to both venous and arterial thromboembolism, including stroke [34]. This hypercoagulable state is characterized by elevated levels of procoagulant factors such as fibrinogen, factor VIII, and von Willebrand factor (vWF), along with reduced fibrinolysis [35]. The pro-thrombotic environment is further enhanced by the activation of platelets and coagulation cascades, along with impaired anticoagulant pathways involving protein C, protein S, and antithrombin III [36]. These factors increase the likelihood of clot formation within cerebral arteries, leading to ischemic stroke [37]. Elevated D-dimer levels, a coagulation activity marker, have been found in IBD patients, particularly during active disease, indicating ongoing clot formation and breakdown [37].

Alterations in Platelet Activity

In IBD, platelets play a pivotal role in inflammation and coagulation [38]. Activated platelets in IBD not only promote thrombus formation but also release pro-inflammatory mediators such as platelet factor 4 (PF4) and soluble cluster of differentiation 40 ligands (sCD40L), which further perpetuate the inflammatory state [38]. Increased platelet activation and aggregation contribute to a hypercoagulable state, and elevated platelet counts (thrombocytosis) are commonly observed in IBD patients during active disease [34]. This combination of platelet activation and aggregation increases the risk of arterial thromboembolism, including ischemic stroke [34,38].

Gut-Brain Axis and Stroke Risk

According to the latest research, the pathophysiology of stroke in IBD is influenced by the gut-brain axis, which emphasizes the reciprocal contact between the central nervous system and the GI tract [30]. Dysbiosis, or the imbalance of gut microbiota, is a common feature in IBD and has been linked to increased systemic inflammation and endothelial dysfunction [39]. Dysbiosis can lead to the translocation of microbial products such as lipopolysaccharides (LPS) into the systemic circulation, triggering inflammatory responses that contribute to gut and vascular inflammation [35]. This systemic inflammatory response may exacerbate vascular injury and increase the risk of ischemic events, including stroke [35].

Impact of Medications on Stroke Risk

**Figure 3:**
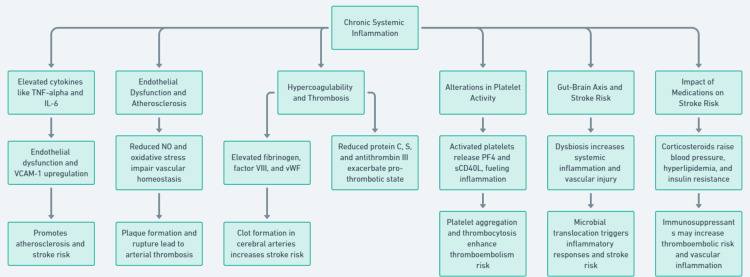
Chronic Systemic Inflammation Linking Inflammatory Bowel Disease to Stroke Risk TNF-α: tumor necrosis factor-alpha; IL-6: interleukin-6; VCAM-1: vascular cell adhesion molecule-1; NO: nitric oxide; VWF: von Willebrand factor; PF4: platelet factor 4; sCD40L: soluble CD40 ligand Image credits: The image has been created by the author Abdallah Khan.

IBD treatment, especially with corticosteroids, has been associated with an increased risk of stroke [30]. Corticosteroids, commonly used to control inflammation in IBD, can cause adverse effects like high blood pressure, hyperlipidemia, and insulin resistance, all of which are recognized risk factors for stroke [30]. Additionally, immunosuppressive agents such as thiopurines and biologics, while reducing inflammation, may alter immune responses and increase the likelihood of thromboembolic incidents [38]. Furthermore, corticosteroid use has been linked to increased vascular inflammation and a pro-thrombotic state, which may explain the higher incidence of cardiovascular events, including stroke, in IBD patients on these medications [34]. These are summarised in Figure 3.

A closer look: studies illuminating stroke risk in IBD

The cohort studies by Keller et al. (2014) [40] and Keller et al. (2015) [41] in Taiwan both examine the relationship between IBD and stroke [40,41]. Keller et al. (2015) [41] studied 3,309 CD patients and reported 919 cases of stroke, finding a heightened risk of stroke in CD patients [41]. In contrast, Keller et al. (2014) [40] investigated 516 UC patients, with 155 reported cases of stroke, emphasizing that stroke risk persists in UC, though less prominently than in CD [40]. Both studies concluded that IBD increases stroke risk [40,41].

The studies conducted by Kristensen et al. (2013) [42] and Dregan et al. (2014) [43] both conducted a cohort study examining stroke risk in IBD patients, but with different focuses [42,43]. Kristensen et al. (2013) [42] involved 24,499 IBD patients, finding 3,781 cases of stroke and a notable link between AF and stroke in IBD patients [42]. However, in their analysis of 19,831 IBD patients, Dregan et al. (2014) [43] found 3,000 occurrences of stroke and highlighted the fact that comorbidities like hypertension raise the risk of stroke [43]. While Dregan et al. (2014) [43] relied on hospital and physician records, which may have captured a more excellent range of comorbid diseases, Kristensen et al. (2013) [42] analysis employed countrywide registries with the International Classification of Diseases, Tenth Revision (ICD-10) codes [44]. Both studies confirmed elevated stroke risk in IBD patients, but Kristensen et al. (2013) focused on the role of AF, whereas Dregan et al. (2014) emphasized the impact of comorbidities [42,43].

Bernstein et al. (2008) [34] and Huang et al. (2014) [45] both explored stroke risk in IBD patients, though they analyzed different populations [34,45]. Bernstein et al. (2008) [34], in a cohort study, analyzed 8,060 IBD patients in Canada, reporting 1,551 stroke cases, indicating an overall increased stroke risk among IBD patients [34]. In comparison, Huang et al. (2014) [45], in a cohort study, examined 18,392 IBD patients in Taiwan and identified 2,533 ischemic stroke cases, focusing specifically on the increased risk of ischemic stroke [45]. The geographical differences between the Canadian and Taiwanese populations might account for the variation in stroke risk, with Huang et al. (2014) [45] study emphasizing ischemic stroke in particular, while Bernstein et al. (2008) [34] findings were broader, covering stroke risk without highlighting specific subtypes [34,45].

These findings confirm the vital link between IBD and increased stroke risk, emphasizing the need for stroke prevention and careful cardiovascular monitoring in IBD patients (Table 1).

**Table 1:** Prevalence of Stroke in IBD Patients CD: Crohn’s disease; UC: ulcerative colitis; IBD: inflammatory bowel disease; ICD-9: International Classification of Diseases, ninth revision; ICD-10: International Classification of Diseases, tenth revision

First Author (Year)	Country	Study Type	Source of Data (Full Form)	No. of IBD Cases (Type of IBD)	No. of Patients Who Developed Stroke (Type of Stroke)	Study Period	Ascertainment of IBD	Ascertainment of Stroke	Conclusion
Bernstein et al. (2008) [34]	Canada	Cohort	Manitoba Health Administrative Database (MHAD)	8,060 (CD/UC)	1,551 (Not specified)	1984-2003	ICD-9 codes [46]	ICD-9 codes [46]	IBD increases stroke risk
										Kristensen et al. (2013) [42]	Denmark	Cohort	Nationwide Registries	24,499 (CD/UC)	3,781 (Atrial fibrillation, stroke)	1996-2011	ICD-10 codes [44]	ICD-10 codes [44]	IBD associated with increased risk of atrial fibrillation and stroke
Keller et al. (2014) [40]	Taiwan	Cohort	Longitudinal Health Insurance Database (LHID2000)	516 (UC)	155 (Not specified)	1995-2005	ICD-9 codes [46]	ICD-9 codes [46]	Stroke risk remains elevated in UC patients										
										Dregan et al. (2014) [43]	United Kingdom	Cohort	Clinical Practice Research Datalink (CPRD)	19,831 (CD/UC)	3000 (Not specified)	2002-2013	Based on medical records and physician diagnosis	Stroke diagnosis using physician and hospital records	IBD associated with increased stroke risk in patients with comorbidities
Huang et al. (2014) [45]	Taiwan	Cohort	National Health Insurance Research Database (NHIRD)	18,392 (CD/UC)	2,533 (Ischemic stroke)	1998-2011	ICD-9 codes [46]	ICD-9 codes [46]	IBD associated with ischemic stroke risk										
										Keller et al. (2015) [41]	Taiwan	Cohort	NHIRD	3,309 (CD)	919 (Not specified)	1995-2005	ICD-9 codes [46]	ICD-9 codes [46]	Increased stroke risk in CD patients

Strategic approaches to IBD management and stroke prevention

A multidisciplinary approach involving gastroenterologists, cardiologists, and other specialists is vital for managing IBD and cardiovascular risks [47]. Effective IBD control requires potent anti-inflammatory therapies [47], while cardiovascular risk factors such as hypertension, dyslipidemia, and diabetes must be carefully managed with pharmacotherapy and lifestyle changes [47]. For high-risk patients, antithrombotic agents may be necessary to reduce thromboembolic and cardiovascular complications [47].

Screening and Management of Traditional Stroke Risk Factors

Considering IBD patients have a higher cardiovascular risk, managing stroke risk factors requires further monitoring, which involves proactive screening and management of hypertension, dyslipidemia, diabetes, smoking, and obesity, as well as optimizing anti-inflammatory therapies [48]. Younger females with IBD have a much higher risk of ischemic stroke, which can last for up to 25 years after diagnosis, underscoring the importance of long-term surveillance [49].

TNF-Alpha Inhibitors to Control IBD Activity

TNF-alpha inhibitors, such as infliximab and adalimumab, effectively reduce systemic inflammation and may lower stroke risk in patients with IBD [50]. TNF-alpha is a key pro-inflammatory mediator, and inhibiting it significantly benefits managing IBD [50]. Infliximab, a potent TNF-alpha blocker, induces apoptosis in TNF-alpha-expressing immune cells and is effective for treating active CD, including fistulas, as well as for UC maintenance therapy [50]. However, infliximab can lead to infusion reactions and autoimmune complications as a foreign protein, necessitating antihistamines and immunosuppressants [50]. Additionally, certolizumab, a humanized anti-TNF-alpha antibody, shows promise for treating CD in ongoing trials [50].

Aspirin and Antiplatelet Therapy

IBD patients who have known cardiovascular risk factors, such as atrial fibrillation (AF) or a history of stroke, should take aspirin or other antiplatelet drugs, such as clopidogrel, to lower their risk of ischemic stroke [51]. Aspirin is routinely used for cardiovascular disease prevention [51]. Moreover, retrospective cohort research revealed that low-dose aspirin did not substantially raise the frequency of IBD exacerbations, suggesting that it might be safe for secondary prevention [51]. Clopidogrel, which irreversibly blocks the purinergic P2Y12 receptor, has shown promise in lowering inflammation in experimental colitis models and appears safe for IBD patients without raising bleeding risk [51]. Thus, low-dose aspirin and clopidogrel may provide cardiovascular protection to IBD patients while reducing flare risks [51].

5-Aminosalicylic Acid (5-ASA) and Statins

Statins lower cardiovascular morbidity and mortality by inhibiting pro-inflammatory mediators, reducing inflammatory activity in IBD [51]. According to studies, atorvastatin may reduce clinical activity in CD and the requirement for corticosteroids, hospitalization, and surgery, while more research is needed to understand these effects fully [51]. 5-ASA therapy in IBD may offer potential benefits in reducing stroke risk [52]. By mitigating systemic inflammation, 5-ASA could play a role in lowering vascular risks associated with IBD, further research is necessary to fully understand the relationship between 5-ASA and stroke risk reduction in IBD [52].

Lifestyle Modifications

Non-pharmacological strategies are essential for managing stroke and cardiovascular risk in IBD patients [53]. Key recommendations include smoking cessation, as it exacerbates disease progression, and regular screening for mental health issues, such as depression and anxiety, with appropriate referrals [53]. Encouraging physical activity, as tolerated, can help reduce inflammation and cardiovascular risk [53]. Dietary interventions should be evidence-based, focusing on inflammation control while addressing obesity and nutritional deficiencies to maintain a healthy body mass index [53]. Omega-3 fatty acids and antioxidant-rich diets are recommended for cardiovascular benefits [53]. Omega-3 fish oil fatty acids (eicosapentaenoic acid (EPA) and docosahexaenoic acid (DHA)) lower leukocyte activity, cytokine production, and inflammatory gene expression, thus lowering inflammation and providing additional benefits in conditions like atherosclerosis and rheumatoid arthritis [53].

Emerging Therapies

Tofacitinib, a Janus kinase inhibitor, is an effective and safe treatment for acute severe ulcerative colitis (ASUC), showing superior clinical remission and endoscopic improvement rates compared to standard care [54]. Patients on tofacitinib experienced faster symptom relief, making it a promising option for rapid induction therapy, particularly in cases refractory to conventional treatments like corticosteroids or biologics [54]. Vitamin D enhances endothelial function and reduces platelet aggregation, potentially lowering the risk of thrombotic events like stroke or heart attack [55]. Deficiency is linked to increased platelet activity, emphasizing its role in cardiovascular health [55]. Maintaining adequate levels may help manage cardiovascular risks, especially in high-risk populations such as hypertensive patients [55].

Surgical Considerations

Patients with severe IBD undergoing colectomy face a substantially higher risk of venous thromboembolism (VTE) during the postoperative period, with those having UC being more susceptible compared to those with CD [56]. The highest incidence of VTE typically occurs within the first two weeks after discharge, underscoring the critical need for sustained thromboprophylaxis throughout hospitalization [56]. Nevertheless, there is limited evidence regarding the effectiveness of thromboprophylaxis after discharge [56]. Prophylactic anticoagulation, typically with low molecular weight heparin (LMWH), should be administered postoperatively to mitigate this risk [56].

Monitoring and Follow-Up

Continuous monitoring of IBD activity and related cardiovascular risk factors is critical for providing effective patient management, especially in lowering the risk of stroke [57]. To optimize treatment options, routine follow-up visits with a multidisciplinary team, including a cardiologist, are recommended [57]. Stable individuals should be evaluated every six to twelve months, but those with active illness may require more regular visits, possibly every three to six months [58]. Furthermore, patients undergoing substantial therapeutic changes or those at high risk of complications may require follow-ups every one to three months [58]. It is critical to tailor the follow-up plan to each patient's specific requirements and circumstances [58].

Future directions

Future research should focus on understanding the molecular mechanisms by which systemic inflammation in IBD causes cerebrovascular damage, including the participation of particular cytokines and inflammatory mediators [35]. The long-term stroke risk in IBD phenotypes must be investigated through longitudinal investigations, including CD and UC, while controlling for disease severity and treatment duration [32]. Furthermore, studies should look at the impact of gut microbiota modulation using probiotics or microbiome-targeted therapeutics on lowering systemic inflammation and stroke risk in IBD patients [59]. Randomized controlled trials are also required to evaluate the efficacy and safety of antithrombotic medicines in preventing cerebrovascular events in high-risk IBD patients [60]. The effect of new IBD medications, such as biologics and small-molecule inhibitors, on cardiovascular outcomes and stroke prevention requires additional exploration [61]. Identifying particular genetic markers related to both IBD and stroke risk may lead to more tailored preventive interventions [62].

Additionally, future research should investigate the effects of lifestyle modifications, including dietary, lifestyle changes, and exercise habits, on stroke risk in IBD populations [63]. Extensive multicenter cohort studies are required to understand how standard cardiovascular risk factors, such as hypertension and diabetes, combine with IBD-specific variables to increase stroke risk [64]. Finally, the impact of IBD-related drugs, such as corticosteroids and immunosuppressants on stroke risk, should be better understood to optimize treatment protocols [43]. Conducting these studies faces challenges such as patient recruitment, funding, and long-term follow-up. Limited access to diverse populations and variability in treatment protocols can also affect results. Addressing these issues is key to obtaining reliable findings.

Limitations

Some research on the association between IBD and stroke risk relies on observational cohort studies, which can introduce several biases, limiting definitive conclusions. Selection bias may occur if certain groups are overrepresented or underrepresented, skewing the results. Additionally, few studies do not differentiate between IBD subtypes (CD vs. UC) or stroke types (ischemic vs. hemorrhagic), leading to classification bias. Variations in study design, patient diversity, and incomplete accounting for confounding factors such as lifestyle and medication use can further impact findings. The evolving use of biologic therapies introduces potential temporal bias, as newer treatments may not be fully captured in long-term studies. These biases underscore the need for further research, particularly randomized controlled trials that account for IBD subtypes, stroke types, and emerging therapies.

## Conclusions

In conclusion, IBD is a significant risk factor for stroke due to chronic inflammation, endothelial dysfunction, and a prothrombotic state. Specific cytokines such as TNF-α and IL-6, along with alterations in gut microbiota, contribute to these pathways, further elevating cerebrovascular risk. Biologics like infliximab and adalimumab, along with statins and antiplatelet therapies such as aspirin, show promise in reducing stroke risk in certain IBD patients. Additionally, non-pharmacological strategies, such as lifestyle modifications, should be considered to provide a more holistic approach to stroke prevention. Future research should focus on personalized therapy techniques, optimizing the timing and combination of interventions, and integrating GI, cardiovascular, and neurological care to reduce stroke rates in this population. This integrated care model will help ensure more effective prevention and management of stroke in IBD patients.
